# Micro 3D Printing of a Temperature-Responsive Hydrogel Using Projection Micro-Stereolithography

**DOI:** 10.1038/s41598-018-20385-2

**Published:** 2018-01-31

**Authors:** Daehoon Han, Zhaocheng Lu, Shawn A. Chester, Howon Lee

**Affiliations:** 1 Department of Mechanical and Aerospace Engineering, Rutgers University, New Brunswick, NJ 08901 USA; 2 Department of Mechanical and Industrial Engineering, New Jersey Institute of Technology, Newark, NJ 07102 USA

## Abstract

Stimuli-responsive hydrogels exhibiting physical or chemical changes in response to environmental conditions have attracted growing attention for the past few decades. Poly(N-isopropylacrylamide) (PNIPAAm), a temperature responsive hydrogel, has been extensively studied in various fields of science and engineering. However, manufacturing of PNIPAAm has been heavily relying on conventional methods such as molding and lithography techniques that are inherently limited to a two-dimensional (2D) space. Here we report the three-dimensional (3D) printing of PNIPAAm using a high-resolution digital additive manufacturing technique, projection micro-stereolithography (PμSL). Control of the temperature dependent deformation of 3D printed PNIPAAm is achieved by controlling manufacturing process parameters as well as polymer resin composition. Also demonstrated is a sequential deformation of a 3D printed PNIPAAm structure by selective incorporation of ionic monomer that shifts the swelling transition temperature of PNIPAAm. This fast, high resolution, and scalable 3D printing method for stimuli-responsive hydrogels may enable many new applications in diverse areas, including flexible sensors and actuators, bio-medical devices, and tissue engineering.

## Introduction

Stimuli-responsive hydrogels are polymeric networks that undergo physical or chemical changes in response to environmental stimuli such as temperature, pH, and electric field^1–3^. Various stimuli-responsive hydrogels and their applications have been explored over the past several decades. Poly(N-isopropylacrylamide) (PNIPAAm) hydrogels, one of the most widely used temperature-responsive hydrogels, have been extensively investigated and used in many applications such as microfluidic devices^4,5^, drug delivery vehicles^6,7^, cell culture substrates^8,9^, and soft actuators^10–13^. PNIPAAm exhibits a large and reversible volume change in water at its Lower Critical Solution Temperature (LCST, typically 32–35 °C) due to a coil-globule transition of the polymer network strands^14,15^. At a temperature below its LCST, NIPAAm molecules in an aqueous environment show a hydrophilic behavior with an extended coil structure, which leads to water uptake and swelling. When the temperature increases above the LCST, however, hydrophobic groups become more active, causing the molecules to transform into a shape resembling a compact globule. Such a dramatic change induces the escape of entrapped water molecules from the hydrogel network, resulting in a significant reduction of volume.

Despite the growing attention to PNIPAAm and its wide range of applications, manufacturing techniques for PNIPAAm have been limited to simple two-dimensional (2D) fabrication methods such as molding and lithography, which impedes full utilization of its unique material behavior. Recently, there have been some efforts on creating a 3D shape from a 2D PNIPAAm sheet using an origami approach^16–18^. However, achieving a high resolution and high aspect ratio PNIPAAm geometry still remains challenging. More recently, additive manufacturing of PNIPAAm has been reported using a commercial extrusion-based 3D printer, but it is still limited to simple 2D extrusion geometries and low resolution^19^. Other high resolution 3D micro-manufacturing techniques including three-dimensional laser chemical vapor deposition (3D-LCVD)^20^, electrochemical fabrication (EFAB)^21^, and micro-stereolithography (μSL)^22^ also have drawbacks such as long fabrication time, high cost, and limited sets of available materials.

**Figure 1:**
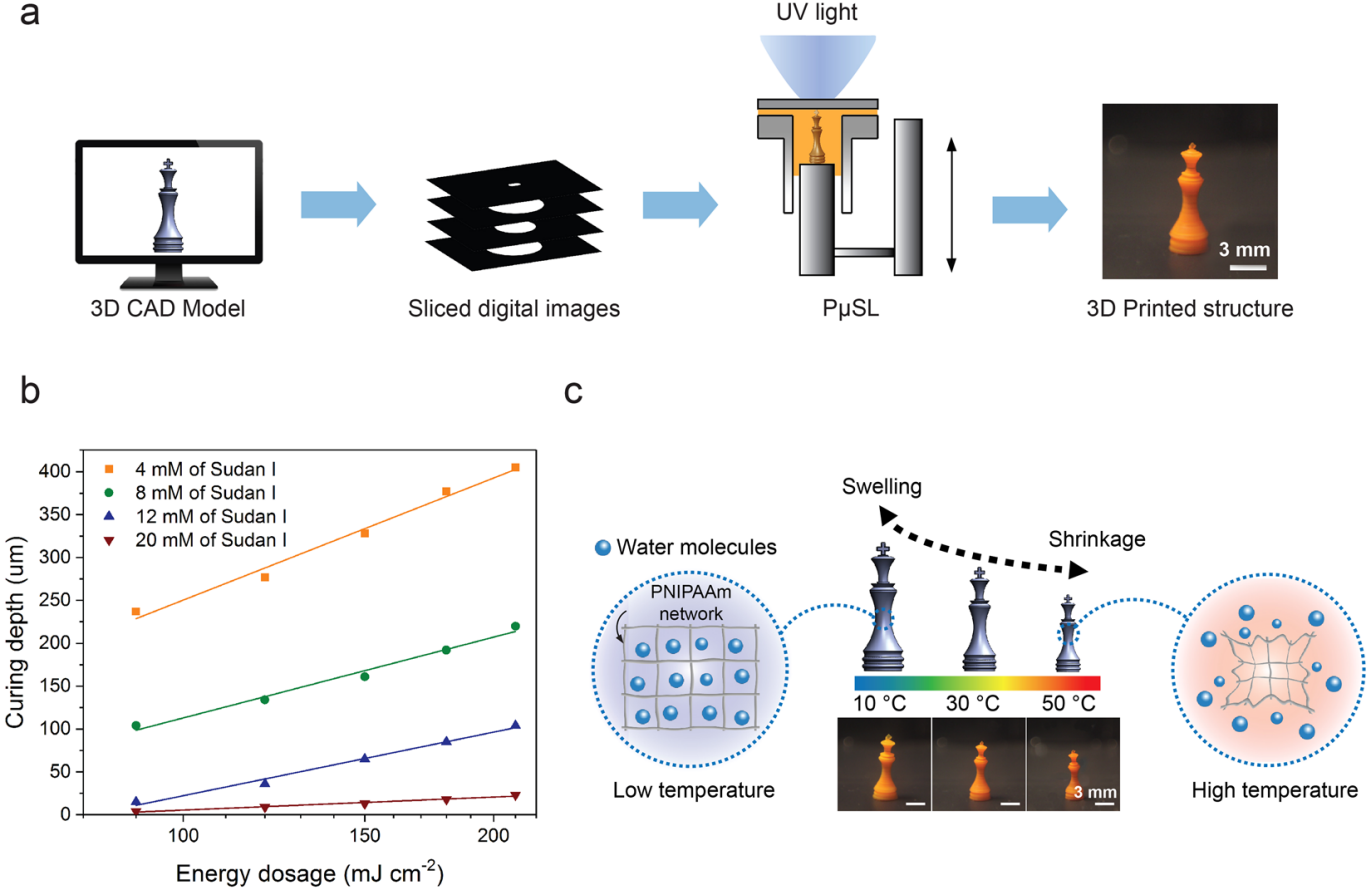
3D printing of temperature responsive hydrogel using PμSL: (**a**) Schematic illustration of the PμSL process. (**b**) The result of curing depth study. Curing depth tends to decrease with increasing PA concentration. Also, higher PA concentration results in slower growth of a layer with increasing energy dosage (The slope of the plot decreases with increasing PA concentration). (**c**) Temperature responsive swelling of 3D printed PNIPAAm hydrogel structure.

In this study, we present high resolution 3D printing of PNIPAAm using projection micro-stereolithography (PμSL). PμSL is a lithography-based additive manufacturing technique that is fast, inexpensive, and flexible in material selection^23,24^. A 3D model is generated using computer-aided-design (CAD) software and digitally sliced into a series of cross-sectional images of the 3D model. Each digital image is transferred to a digital mask to optically pattern ultra-violet (UV) light, which is then projected through a reduction lens and focused on the surface of photo-curable resin. The patterned UV light converts liquid resin to a solid layer through photo-polymerization. Once a layer is formed, the linear stage drops the sample holder on which the object is built in order to introduce fresh liquid resin for the next layer. The subsequent layer is polymerized in the same manner on top of the preceding layer. This process is repeated until all the layers are complete to build the 3D object (Fig. 1a and Supplementary Fig. S1).

Since PμSL uses projection lithography, an entire layer is polymerized by a single UV illumination of a few seconds. Therefore, fabrication speed is much faster than serial processing techniques where a nozzle or a laser beam has to be raster-scanned for each layer. Also, the use of a reconfigurable digital photo mask eliminates the need for multiple physical photo masks which would otherwise make the fabrication process very expensive and time-consuming. PμSL is also compatible with various photo-curable materials including poly (ethylene glycol) (PEG), poly lactic acid (PLA), poly(caprolactone) (PCL), and their copolymers^23–27^. In this work, we demonstrate the 3D printing of PNIPAAm using PμSL and the reversible deformation of various 3D printed PNIPAAm micro-structures. We also investigate the effects of PμSL process parameters as well as polymer resin composition on the temperature dependent swelling behavior of 3D printed PNIPAAm hydrogel. Also demonstrated is the sequential deformation of a 3D printed PNIPAAm structure by selective incorporation of an ionic monomer that shifts the swelling transition temperature of PNIPAAm.

## Results and Discussion

### 3D Printing of PNIPAAm

All photo-curable PNIPAAm resins used in this work were prepared by dissolving NIPAAm as a monomer, N,N'-Methylene-bis(acrylamide) as a cross-linker, and Phenylbis (2,4,6-tri-methylbenzoyl) phosphine oxide as a photo-initiator (PI) that initiates a photo-polymerization upon light absorption, all of which were dissolved in ethanol as a base solvent. In addition, Sudan I was added in the resins as a photo-absorber (PA) that controls the penetration depth of light. Rhodamine B was also added as a dye for visualization of 3D printed PNIPAAm structures. Table 1 provides the chemical components of the photo-curable resins used in each study and their concentrations. In addition, the exact quantities of the components in the resins are provided in Supplementary Table S1.

**Table 1:** Chemical composition of different polymer resins.

Study	Chemical composition						
	NIPAAm (M)	Cross-linker (mM)	Molar ratio of cross-linker to monomer	PI (mM)	PA (mM)	Rhodamine B (mM)	MAPTAC (M)
Curing depth (Section 2.1)	6.2	324	0.05	47.8	4	2.1	—
					8		
					12		
					20		
Cross-linker (Molar ratio) (Section 2.2.1)	6.2	65	0.01	47.8	12	2.1	—
		130	0.02				
		195	0.03				
		259	0.04				
		324	0.05				
NIPAAm concentration (Section 2.2.1)	2.6	139	0.05	20.5	12	2.1	—
	4.4	232		34.1			
	6.2	324		47.8			
	8.0	417		61.4			
	9.7	510		75.1			
Light intensity (Section 2.2.2)	6.2	324	0.05	47.8	12	2.1	—
Layer thickness (Section 2.2.3)	6.2	324	0.05	47.8	12	2.1	—
Ionic monomer, MAPTAC (Section 2.2.4)	6.2	324	0.05	47.8	12	2.1	—
							0.2
							0.4
Gripper (Section 2.3)	6.2	324	0.05	47.8	12	2.1	—
Dumbbell (Section 2.3)	6.2	259	0.04	47.8	12	2.1	—
		324	0.05				0.4

Ethanol was used as a solvent for all solutions.

Lateral resolution of the PμSL system is determined by the optics of the PμSL system and the polymerization kinetics of PNIPAAm. With the current PμSL system and the PNIPAAm resin, a lateral resolution of around 160 μm was achieved for PNIPAAm hydrogel (Supplementary Fig. S2). In order to build a 3D object in a layer-by-layer fashion, we also studied how a layer of PNIPAAm hydrogel is polymerized and grows in the vertical direction. The depth of photo-polymerization, or curing depth, was measured as a function of light energy dosage. To study the curing depth, rectangular images were projected through a transparent glass slide into PNIPAAm resin for different curing times (Supplementary Fig. S3). Since polymerization begins from the surface of the glass slide and grows with exposure time, the curing depth can be obtained by measuring the height of resulting rectangular pattern using atomic force microscope (AFM). The same experiment was repeated with PNIPAAm resins having different concentration of PA. As shown in Fig. 1b, the curing depth of PNIPAAm hydrogel can be prescribed by controlling the light energy dosage and PA concentration in the resin.

Based on the process characteristics determined by the above experiments, 3D printing of PNIPAAm was demonstrated as shown in Fig. 1c. The height of the object is 7.83 mm, consisting of 261 layers having a thickness of 30 μm. A nine second curing time was used for each layer, making the total fabrication time of 65 min. The smallest lateral feature size of the object is 163 μm. After printing, the PNIPAAm micro-structure was stored in DI water at 15 °C overnight for post-printing rinsing. During that time, the ethanol inside the structure is replaced with DI water, and uncross-linked polymers and other chemical components in the resin are removed from the printed structure. Therefore, no further cross-linking occurs with time after rinsing. To confirm the temperature dependent deformation of the printed structure, it was put into a temperature-controlled water chamber while the deformation of structure was recorded (Supplementary video S1). As shown in Fig. 1c, the 3D PNIPAAm hydrogel structure was swollen to a height of 9.63 mm at 10 °C, which then shrank to a height of 7.41 mm when the temperature was raised to 50 °C. The rate of temperature increase and that of temperature decrease were 1.33 °C/min and 0.67 °C/min, respectively. Reversible swelling deformation was observed when the temperature was dropped to 10 °C again, demonstrating that swelling deformation of a 3D PNIPAAm hydrogel printed by PμSL can be controlled by environmental temperature.

### Controlling temperature responsive swelling of PNIPAAm hydrogel

The key attribute of PNIPAAm hydrogel is its ability to change its degree of swelling in response to temperature change. To quantify this, we use the swelling ratio (SR) in this study defined as a ratio of swollen length to the original length (as fabricated). It is important to note that the reference dimension is not the dimension of the hydrogel in the dry state, but the original dimension at the moment of polymerization (or printing). For example, the SR is greater than 1 when a PNIPAAm hydrogel swells and gets bigger than the printed dimension at low temperature, and the SR is less than 1 when it shrinks and becomes smaller than the printed dimension at high temperature.

**Figure 2:**
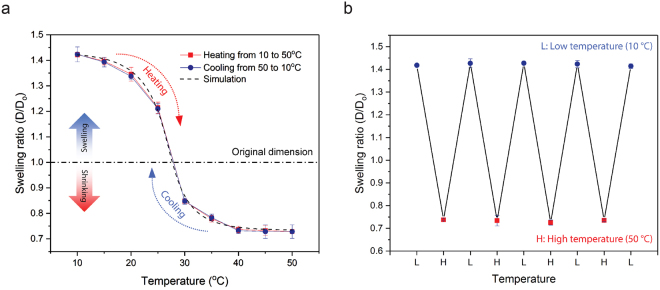
Reversible temperature dependent swelling/shrinkage of PNIPAAm hydrogel. (**a**) Equilibrium swelling of PNIPAAm depends on temperature. Swelling ratios of PNIPAAm during a heating cycle match well with those from a cooling cycles. Also, experimental results are in a good agreement with the equilibrium swelling simulation result. (**b**) Low temperature swelling and high temperature shrinkage of PNIPAAm occurred repeatedly when heated and cooled alternately between 10 °C and 50 °C.

To observe the overall swelling behavior of PNIPAAm in response to temperature, disk-shaped samples with a diameter of 1.6 mm and a thickness of 300 μm were printed using PμSL. The NIPAAm and cross-linker concentrations of the polymer resin used in this study were 2.6 M and 139 mM in ethanol, respectively. As shown in Fig. 2a, swelling ratio at a low temperature of 10 °C is 1.42. It shows a 42% increase in length (or 186% in volume) compared to the original dimensions of the printed disk sample. On the other hand, the swelling ratio is 0.73 at a high temperature of 50 °C, which indicates a 27% decrease in length (or 61% in volume) from the printed dimension of the sample. Furthermore, the swelling ratio was measured at every 5 °C while increasing temperature from 10 °C to 50 °C and subsequently decreasing temperature back to 10 °C. The temperature was changed by 5 °C every 3 hours to ensure swelling reaches an equilibrium state. The result shows that swelling ratios in the heating and cooling cycles are well-overlapped. Also, the maximum and minimum swelling ratios remain constant after multiple cycles of heating and cooling (Fig. 2b), showing that temperature dependent swelling of PNIPAAm is reversible and repeatable. Thermally responsive gel behavior is analyzed in the context of the theoretical model in the recent study^28^, showing good agreement with experimental data (Fig. 2a) (See Supplementary information for details).

Thermodynamically, the equilibrium swelling ratio of a gel in a solvent is governed by the cross-linking density of the polymer network and its interaction with that solvent. Since we use photo-polymerization to cure PNIPAAm hydrogels using PμSL, the temperature responsive swelling behavior of 3D printed PNIPAAm hydrogel may be tailored by controlling polymerization kinetics in the PμSL process. In this study, some key parameters including polymer resin composition, curing light intensity, and layer thickness of 3D printed PNIPAAm are studied.

#### Effect of polymer resin composition on temperature responsive swelling

The swelling behavior of hydrogels is closely associated with the polymer chain length; for instance, a hydrogel comprised of long polymer chains exhibits a large degree of swelling^29^. Since the polymer network of a PNIPAAm hydrogel is formed by the cross-linkers that connect NIPAAm monomers, the polymer chain length is determined by molar ratio of cross-linker to NIPAAm monomer. In other words, a high ratio of cross-linker to monomer results in more cross-linking sites and therefore a shorter polymer chain length, leading to a low degree of swelling. The overall concentration of monomer also affects the swelling behavior because it determines the volume of a shrunken PNIPAAm hydrogel at high temperature. To confirm the effect of the molar ratio of cross-linker to monomer on swelling behavior, five different disk shaped samples were printed by PμSL. All of the samples were created with the same monomer concentration, but different molar ratios, ranging from 0.01 to 0.05 (Table 1). Since the concentration of NIPAAm is significantly higher than that of cross-linker throughout the five samples, the sum of monomer and cross-linker molecules in all samples were approximately the same.

**Figure 3:**
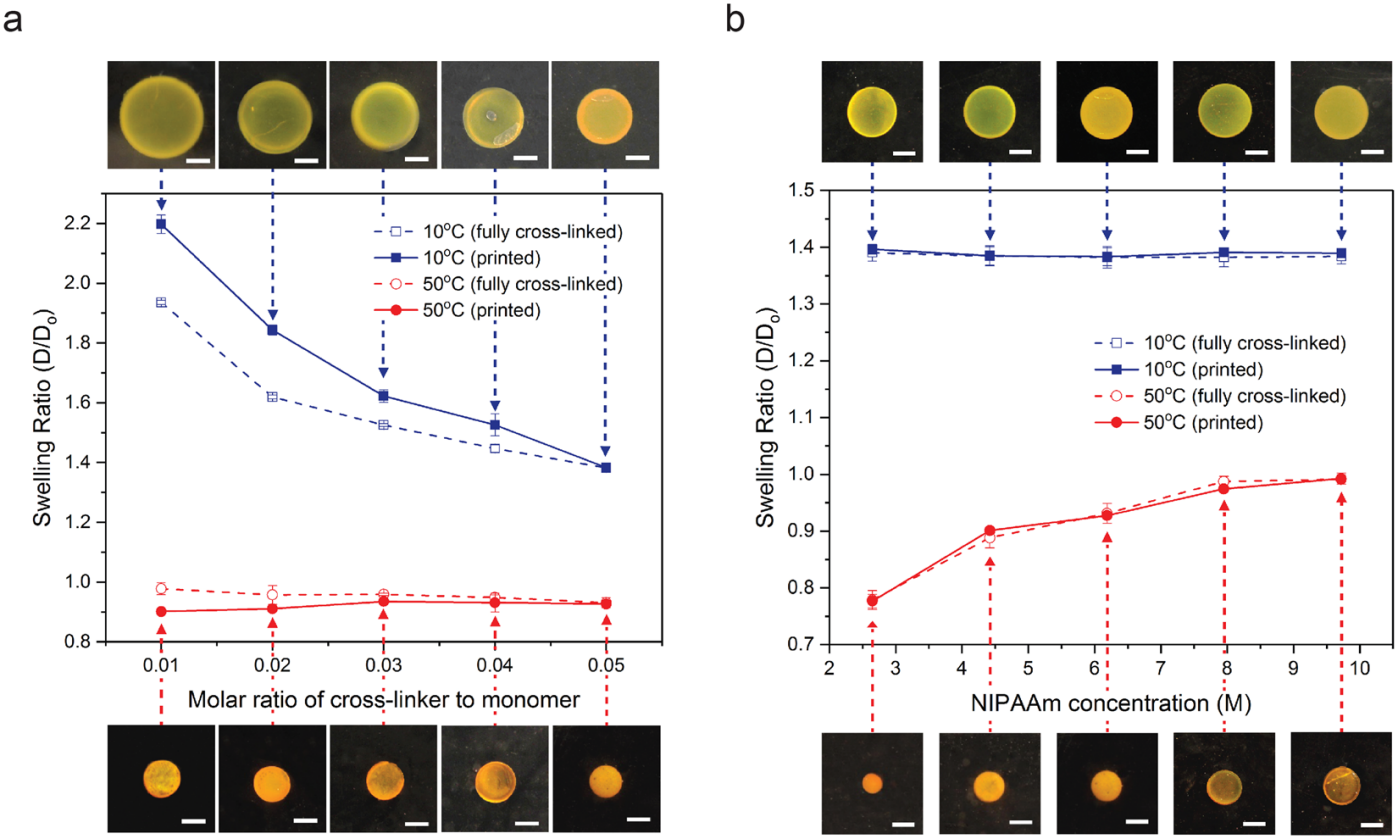
Effects of the chemical composition of photo-curable resin on temperature responsive swelling of PNIPAAm. (**a**) Low temperature swelling of PNIPAAm is determined by the molar ratio of cross-linker to NIPAAm monomer. (**b**) High temperature shrinkage of PNIPAAm is determined by the NIPAAm concentration. Solid and dashed lines are for the samples fabricated using PμSL and a UV oven, respectively. Blue and red lines are for the swelling ratio at 10 °C and 50 °C, respectively. The photo images show the size of corresponding PNIPAAm disk samples at 10 °C and 50 °C. All scale bars indicate 1 mm.

As shown in Fig. 3a, samples with different molar ratios exhibit different swelling behavior at low temperature. It proves that a low molar ratio creates long polymer chains that can stretch further, leading to a high swelling ratio at low temperature (10 °C, blue solid squares in Fig. 3a) as described in the earlier sections. By lowering the molar ratio from 0.05 to 0.01, approximately a 60% increase in swelling ratio was achieved at 10 °C. On the contrary, the swelling ratio at high temperature (50 °C, red solid circles in Fig. 3a) remained the same around 0.9 across all samples regardless of molar ratio. This is because the concentration of monomer molecules in the resin determines the polymer volume fraction of the cross-linked hydrogel. The swelling of the fully cross-linked samples prepared by a UV oven with a significantly higher exposure dosage (6000 mJ cm^−2^) showed the similar trend (open squares and circles in Fig. 3a). Swelling differences between printed samples and UV oven cured fully cross-linked samples at low temperature is attributed to low reactivity of resins with low cross-linker concentration. When a sample was cured in a UV oven, we applied a high energy dose of 6000 mJ cm^−2^ such that all cross-linker molecules in the resin can form cross-links regardless of the reactivity of the resin. However, for 3D printed samples, an energy dose of 300 mJ cm^−2^ was given to all five printed samples to ensure printing quality and to avoid over-curing (Supplementary Table S2). Therefore, the cross-linking density of printed samples with low cross-linker concentrations is lower than that of UV oven cured fully cross-linked samples, leading to higher swelling in low temperature. This trend becomes more apparent for the samples with lower cross-linker concentrations. On the other hand, when the cross-linker concentration reaches a ratio of 0.05 to NIPAAm monomer, the reactivity of the resin is high enough that the cross-linking density of the printed sample reaches that of the UV oven cured counterpart. Therefore, we can conclude that swelling of 3D printed PNIPAAm at low temperature can be prescribed by controlling the ratio of cross-linker with respect to NIPAAm in the resin.

To study the effect of the monomer concentration on the swelling behavior, we prepared five samples with different NIPAAm concentrations, 2.6, 4.4, 6.2, 8.0, and 9.7 M, respectively, while keeping the cross-linker to monomer ratio fixed at 0.05. Figure 3b shows that NIPAAm concentration causes significant change in shrinkage at high temperature, while its influence on low temperature swelling is negligible. By decreasing the NIPAAm concentration from 9.7 to 2.6 M, approximately a 20% difference in the swelling ratio was achieved at 50 °C. It also showed very good agreement with the results obtained from similar fully cross-linked samples prepared using the UV oven (the open squares and circles). Since the rate of polymerization is strongly dependent on monomer concentration, a different curing time was used for a different NIPAAm concentration (Supplementary Fig. S4 and Supplementary Table S2).

#### Effect of curing light intensity of PμSL on temperature responsive swelling

A light exposure intensity determines the rate of photo-polymerization, which in turn determines the cross-linking density of the hydrogel^30^. The cross-linking density is the most important factor that determines the physical properties of the polymer network, including the equilibrium swelling ratio^29^. Since the PμSL system employs a digital image to generate a pattern, the projection light intensity can be easily tuned by controlling the grayscale value of the digital image^31,32^ (Supplementary Fig. S5). The grayscale value of a typical 8-bit digital image ranges from 0 to 255, with 0 being black and 255 being white.

**Figure 4:**
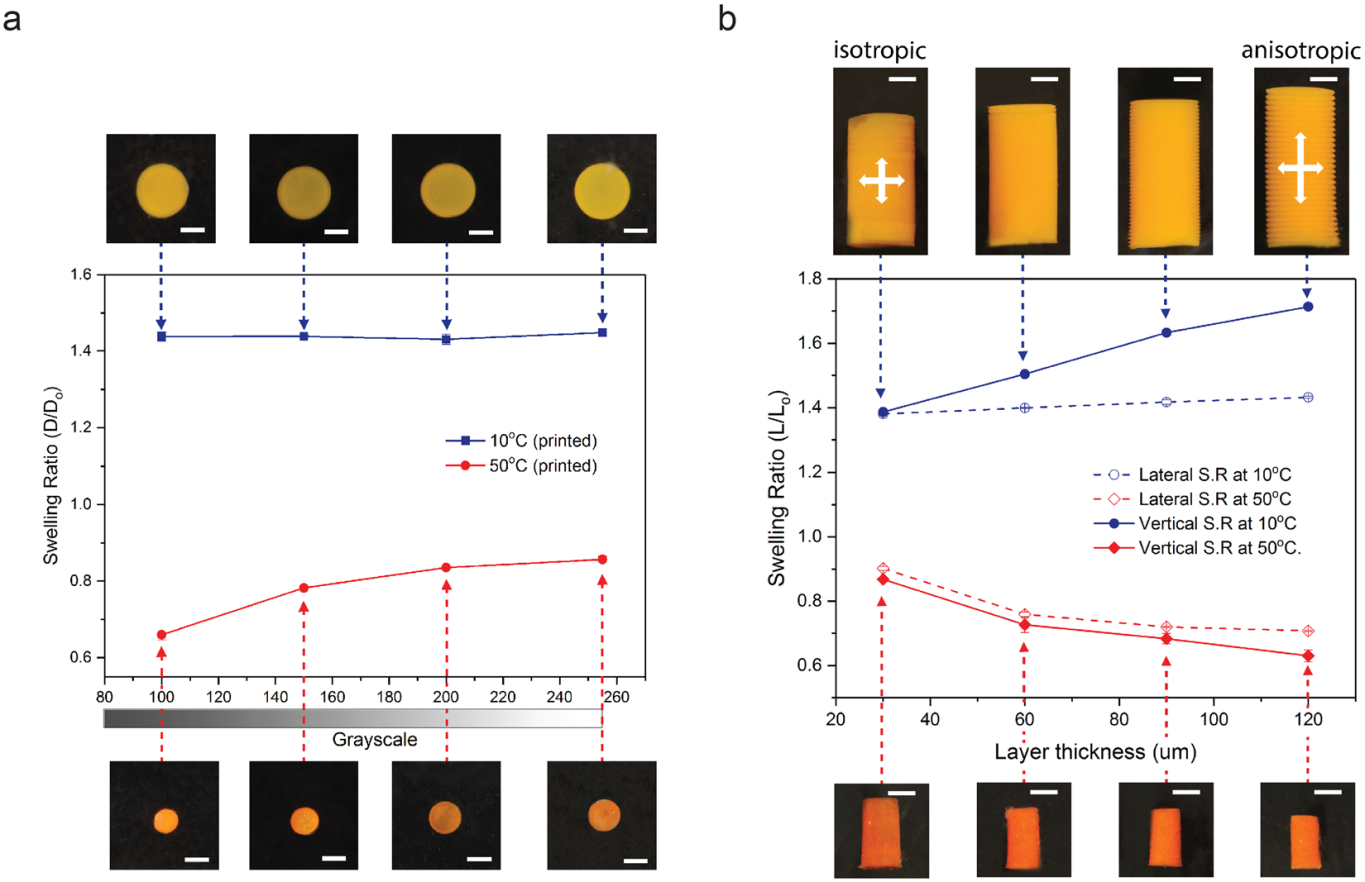
Control of temperature dependent swelling of PNIPAAm using PμSL process parameters. (**a**) As the grayscale level of a projected digital image decreases from white (gray scale of 255) to black (gray scale of 0), the swelling ratio at high temperature of PNIPAAm hydrogel is significantly reduced. Blue and red lines are for the swelling ratio at 10 °C and 50 °C, respectively. (**b**) Vertical swelling at low temperature increases with layer thickness, while lateral swelling is independent of layer thickness. Shrinkage at high temperature is relatively insensitive to layer thickness. Solid and dashed blue lines are for the swelling ratio at 10 °C in vertical and lateral directions, respectively. Solid and dashed red lines are for the swelling ratio at 50 °C in vertical and lateral directions, respectively. The photo images show the size of corresponding PNIPAAm disks (top view) and cylinders (side view) at 10 °C and 50 °C. All scale bars indicate 1 mm.

To study the effect of curing light intensity on the degree of swelling, four different disk-shaped samples were fabricated by PμSL using digital images with grayscale values of 100 (4.3 mW cm^−2^), 150 (7.3 mW cm^−2^), 200 (14.3 mW cm^−2^), and 255 (30.0 mW cm^−2^) (Supplementary Table S2). Figure 4a shows that the swelling ratio of the 3D printed structures at low temperature (10 °C, blue solid square in Fig. 4a) remains constant at 1.43, whereas the swelling ratio at high temperature (50 °C, red solid circle in Fig. 4a) depends on the grayscale value. PNIPAAm samples printed using a grayscale value of 100 shrank to a swelling ratio of 0.66 at 50 °C while those printed using a white image resulted in a swelling ratio of 0.86 at the same temperature, showing an approximately 23% difference in the swelling ratio. This trend is similar to the results from the study of monomer concentration presented in the previous section (Fig. 3b), which implies that the amount of molecules cross-linked into a polymer network decreases with a decreasing light intensity modulated by a grayscale level of the digital image. This is because lower irradiation energy leaves more NIPAAm monomers in the resin uncross-linked, which are later rinsed out from the polymer network by the post-printing rinsing process. Similarly, the low temperature swelling ratio is the same throughout all samples because they were all printed using the same resin (Table 1). This result demonstrates that temperature dependent swelling can also be controlled using PμSL even without changing the resin. Furthermore, non-uniform swelling may also be programmed within a layer simply by designing an appropriate distribution of grayscale in the projected digital image.

#### Effect of layer thickness of 3D printed PNIPAAm on temperature responsive swelling

PμSL, like other 3D printing techniques, builds a 3D object in a layer-by-layer fashion. When each layer is solidified, a UV light is focused on the surface of the resin from which photo-polymerization is initiated and gradually propagates into the resin. Hence, within a layer, a gradient of cross-linking density is created with the upper part being highly cross-linked. Consequently, the swelling behavior throughout the thickness is not uniform because it strongly depends on the cross-linking density as studied on the previous sections (Supplementary Fig. S6a). As the difference in cross-linking density across the thickness of a layer increases with layer thickness, the bottom portion of a relatively thick layer tends to have a relatively lower cross-linking density and swell further as a consequence (Supplementary Fig. S6b). When these layers are stacked to create a 3D PNIPAAm structure, the relatively lower cross-linked bottom portion of a layer is bonded to the highly cross-linked top portion of the layer underneath. Due to this physical constraint at the interface, the amount of lateral swelling is therefore dominated by the highly cross-linked upper portion of each layer. On the other hand, the layers can swell freely in the vertical direction without any constraint. (Supplementary Fig. S6c). As a result, it is possible to create anisotropy in temperature responsive swelling of 3D printed PNIPAAm hydrogel by controlling a layer thickness in the PμSL process.

To study the effect of layer thickness on the lateral and vertical swelling of PNIPAAm, cylindrical samples were printed with the same resin (Table 1) but different layer thicknesses (Supplementary Table S2). As shown in Fig. 4b, the sample fabricated with a 30 μm layer thickness showed the same swelling ratios in both lateral and vertical directions at high and low temperatures. This implies that cross-linking density within these thin layers is relatively uniform. However, as the layer thickness increases, the amount of vertical swelling at low temperature increases significantly (blue solid circle in Fig. 4b) while lateral swelling remains almost identical regardless of layer thickness (blue open circle in Fig. 4b). When the PNIPAAm hydrogel shrinks at high temperature, the deformation in both lateral (red open diamond in Fig. 4b) and vertical (red solid diamond in Fig. 4b) directions were similar regardless of layer thickness. This result demonstrates that PμSL provides a unique capability to create anisotropy in thermo-responsive swelling of PNIPAAm hydrogel.

#### Effect of ionic co-monomer on temperature responsive swelling

**Figure 5:**
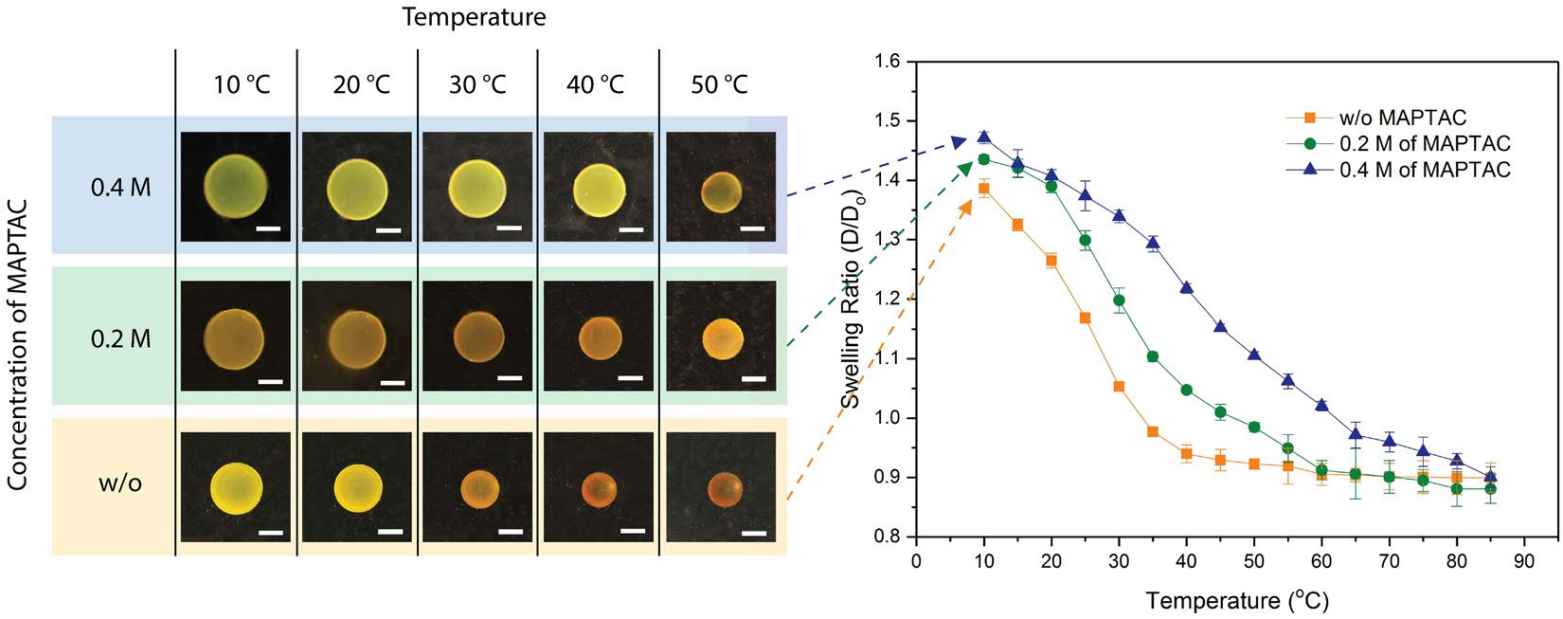
The swelling transition temperature of PNIPAAm increases by adding ionic co-monomer. Green and blue lines show temperature responsive swelling of PNIPAAm with 0.2 M and 0.4 M of MAPTAC, respectively. The swelling ratio of the standard PNIPAAm without MAPTAC (orange line) was measured as a control. The photo images show the size of corresponding disk samples at different temperatures. All scale bars indicate 1 mm.

The transition temperature of 3D printed PNIPAAm structures is the LCST of NIPAAm, 32–35 °C. However, it can be shifted by adding positively charged ionic co-monomer, Methacrylamidopropyltrimethyl-ammonium Chloride (MAPTAC), to the polymer resin. The positively charged MAPTAC integrated in the cross-linked network increases hydrophilicity of a polymer chain because of the growing ratio of cationic site^33,34^. As a result, the transition temperature shifts up from around 32 °C to a higher temperature. To confirm the effect of MAPTAC, three different PNIPAAm disks were printed with different concentrations of MAPTAC (Table 1 and Supplementary Table S2). As shown in Fig. 5, the disk without MAPTAC dramatically changes in size between 20 and 30 °C (orange box and line in Fig. 5), whereas adding 0.2 and 0.4 M of MAPTAC resulted in shift in transition temperature to 30–40 °C (green box and line in Fig. 5) and 40–50 °C (blue box and line in Fig. 5), respectively. The temperature at which more than 80% of the entire swelling deformation was achieved shifts from 35 °C for original PNIPAAm to 50 °C and 65 °C for 0.2 M and 0.4 M of MAPTAC, respectively (Supplementary Fig. S7). Furthermore, adding MAPTAC to PNIPAAm allows for broadening and linearizing the swelling transition, which could potentially be utilized in various sensing applications.

### 3D printed PNIPAAm micro-structures

**Figure 6:**
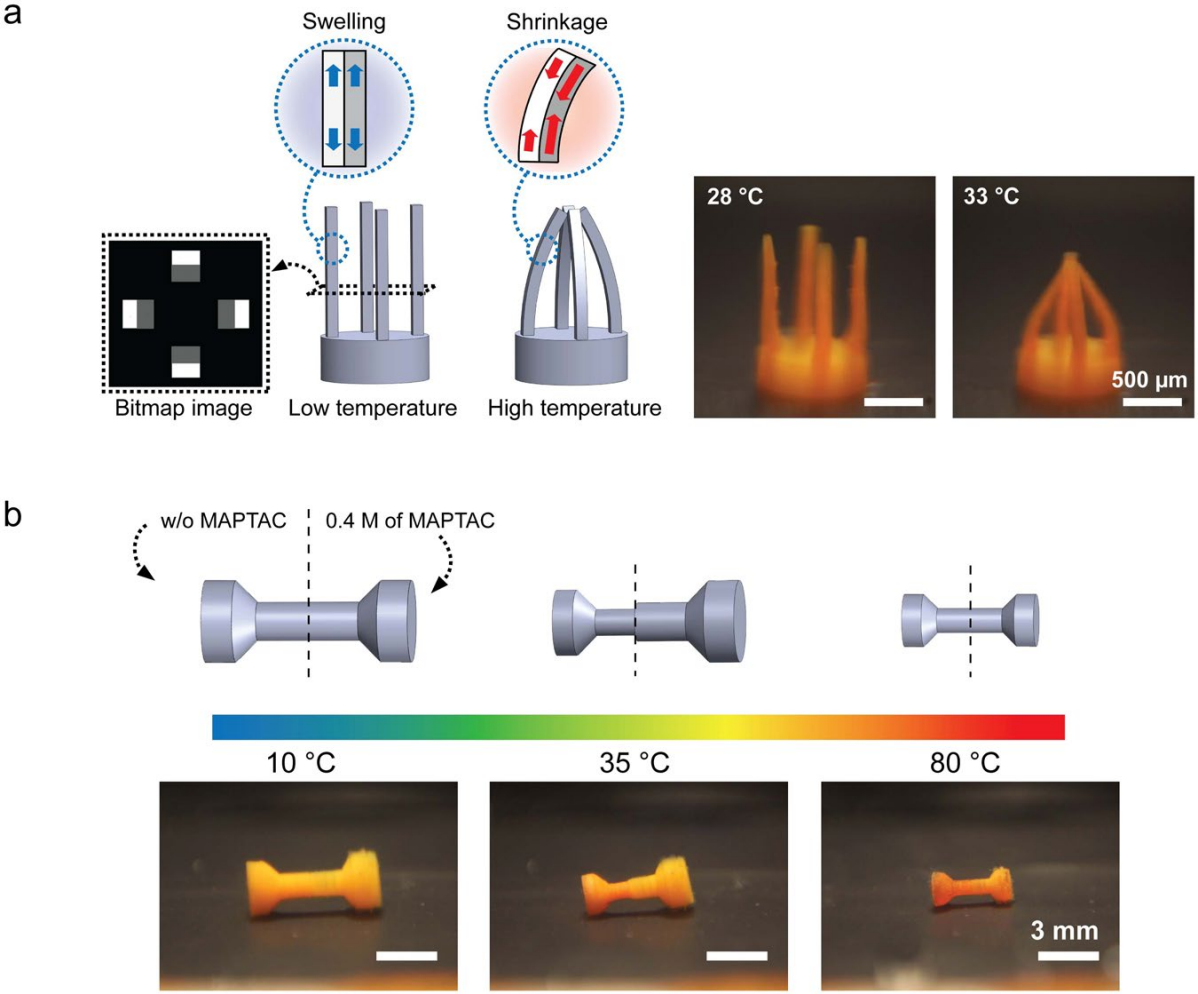
3D printed PNIPAAm micro-structures and their programmed temperature dependent deformation. (**a**) A gripper consisting of four beams was fabricated using two different grayscale levels. The difference in the swelling ratio between the two regions caused the beams to bend towards the center at high temperature (scale bar: 500 μm). (**b**) A dumbbell-shaped structure was printed with ionic monomer, MAPTAC. The left half is pure PNIPAAm while the right half contains 0.4 M of MAPTAC. When temperature increases, the left half with lower transition temperature begins to shrink first, and the right half shrinks later at higher temperature (scale bar: 3 mm).

Based on the findings presented above, we 3D printed PNIPAAm structures using a variety of different process parameters. Figure 6a shows a gripper consisting of four beams fabricated using two different levels of grayscale. Due to the different levels of light intensity (4.3 mW cm^−2^ for gray (100) and 30 mW cm^−2^ for white (255)), a virtual bilayer beam having two different swelling characteristics was created. Different swelling ratios in different regions of the cross-section generated a strain mismatch between the two regions, causing a bending deformation of the beam as the temperature is cycled. At temperatures below the transition temperature, all beams are straight since both white and gray regions have a similar swelling ratio. As temperature increases, the gray regions facing the center start to shrink more than white regions, causing the beams to bend inward to generate a gripping motion (Supplementary video S2). When the temperature lowers, the beams become straight again. Also, a dumbbell-shaped structure in Fig. 6b was fabricated using two different resins having different transition temperatures. The left half was made of pure PNIPAAm and the right half was made using a PNIPAAm resin with 0.4 M of MAPTAC. At low temperature (10 °C), both sides are fully swollen, so the dumbbell is symmetric. As the temperature increases, the left half begins to shrink while the right half remains in its fully swollen state, posing an asymmetric shape at a temperature near 35 °C. When temperature further increases beyond the transition temperature of the right half, shrinking starts to occur in the right half as well, and the dumbbell eventually becomes symmetric again, but in a smaller shrunken dimension (Supplementary video S3). As demonstrated here, PμSL provides a unique fabrication capability to easily program complex temperature responsive deformation in 3D PNIPAAm micro-structures.

## Conclusion

We presented the 3D fabrication of PNIPAAm micro-structures using high resolution PμSL. The effects of various material and process parameters involved in the PμSL process on the thermally responsive swelling of 3D printed PNIPAAm were investigated. We found that PNIPAAm swelling at low temperature and shrinking at high temperature can be independently controlled. The molar ratio of cross-linker to monomer determines the low temperature swelling of PNIPAAm, while high temperature shrinking of PNIPAAm is primarily determined by the monomer concentration. Furthermore, we demonstrated that grayscale of digital images can be utilized to control curing light intensity, which mainly affects the high temperature shrinkage of 3D printed PNIPAAm. This indicates that temperature dependent swelling behavior of PNIPAAm can be spatially encoded and distributed within a layer without the need for time- and material-consuming resin exchange. We have also shown that 3D printed PNIPAAm swelling in the lateral and vertical directions is dependent on the layer thickness, through which anisotropic thermo-responsive swelling can be achieved by design. In addition, control of the swelling transition temperature of PNIPAAm was demonstrated by incorporating a hydrophilic ionic monomer. The transition temperature was increased with increasing ionic monomer concentration. Overall, we believe that the presented method of using PμSL to three-dimensionally fabricate PNIPAAm micro-structures, along with the understanding of the temperature responsive swelling characteristics of PNIPAAm, will significantly extend the potential of smart materials for various applications, such as soft robots, microfluidic devices, and drug delivery vehicles.

## Materials and Methods

### Materials

The resin used in this study consists of N-isopropylacrylamide (NIPAAm) (Fisher Scientific), N,N'-Methylene-bis(acrylamide) (Sigma-Aldrich) as a cross-linker, Phenylbis (2,4,6-tri-methylbenzoyl) phosphine oxide (Sigma-Aldrich) as a PI, Sudan I (Sigma-Aldrich) as a PA, and a fluorescent dye Rhodamine B (Sigma-Aldrich). An ionic monomer Methacryl-amidopropyl-trimethyl-ammonium Chloride (MAPTAC) (Sigma-Aldrich) was used for the transition temperature study. Ethanol was used as a solvent in all resins. All materials were used as received.

### Projection Micro-StereoLithography (PμSL)

A custom-made PμSL system was built using the following major components; Liquid crystal on silicon (LCoS) as a digital dynamic mask, a projection lens (Carl Zeiss), a UV LED (405 nm, Innovations in Optics), a linear stage (Newport Corporation), and collimation optics (Thorlabs). An LCoS digital dynamic mask was extracted from a commercial digital projector (Cannon).

### Curing depth study

To prepare the photo-curable resins for curing depth study, 6.2 M of NIPAAm was dissolved in ethanol with 324 mM of cross-linker, 47.8 mM of PI, and 2.1 mM of Rhodamine B. Then, 4, 8, 12, or 20 mM of Sudan I was added to the resin as a PA to confirm the effect of PA concentration on curing depth. The prepared resin was filled into a glass mold which has 1 mm thickness, and the rectangular images were projected with 30.0 mW cm^−2^ of light intensity through a transparent glass mold into PNIPAAm resin for different curing times (Supplementary Fig. S3). Due to the curing time difference, rectangular structures of different heights were grown from the glass surface. To measure the height of the structures on glass slide precisely, an AFM (NX-10, Park systems Corp.) was used in contact mode.

### Sample fabrication using a UV oven

The photo-curable resins were prepared without PA and Rhodamine B to fabricate a fully cross-linked structure. The resins were cured with more than enough light energy (6000 mJ cm^−2^) using a UV oven (CL-1000L, UVP, 365 nm). First, thin films of PNIAAm were cured with a transparent glass mold having a 360 μm thickness. PNIPAAm disks were made from these films using a punch tool with a diameter of 4.8 mm. The disks were rinsed in DI water at 15 °C overnight.

### Temperature responsive swelling characterization

Based on the curing depth study, the photo-curable resin which has 6.2 M of NIPAAm, 324 mM of cross-linker, 47.8 mM of PI, 12 mM of PA, and 2.1 mM of Rhodamine B was chosen as a base resin. In addition, a light intensity of 30.0 mW cm^−2^, curing time of 10 sec, and layer thickness of 30 μm, were chosen as standard process parameters. To study the effect of polymer resin composition, two or three chemical concentrations were modified for each study. In addition, 0.2 and 0.4 M of MAPTAC were added to the base resin to study the effect of ionic monomer on transition temperature (Table 1). The light intensity and layer thickness were controlled to study the effect of PμSL process parameters on swelling of 3D printed PNIPAAm (Supplementary Table S2). The cylindrical samples with a diameter of 1.6 mm and a height of 3.6 mm were printed for the layer thickness study, and disk samples of a diameter of 1.6 mm and a thickness of 300 μm were printed for the rest of the swelling characterization.

### Post-printing rinsing process

After fabrication, the samples were rinsed and stored in DI water at 15 °C overnight in order to exchange the ethanol and remaining uncross-linked polymers in the network with DI water. Typical volume of the DI water used in rinsing is 60 ml for a sample having a volume of about 6 × 10^−4^ cm^3^. At this low temperature of 15 °C, PNIPAAm becomes hydrophilic and thus allows water molecules to diffuse into the cross-linked network to replace ethanol in the network. With an abundant amount of rinsing water, ethanol in the printed PNIPAAm is effectively diluted and replaced with water, which results in large swelling of PNIPAAm.

### Swelling ratio measurement

The swelling ratio of PNIPAAm samples was obtained by optically measuring the diameter or length of the structure. After fabrication, all the samples were stored in DI water at 15 °C overnight for post-printing rinsing. Then the samples were put into a temperature-controlled chamber filled with DI water. The chamber has a transparent glass window through which temperature dependent deformation of samples were observed and measured. The temperature of water in the chamber were controlled within a range from 10 °C to 90 °C. The rate of temperature change was 0.4 °C/min. The photo images of structures were taken at every 5 °C using a digital camera coupled with a microscope objective lens. Sample dimension was measured from the digital images using image analysis software, Image J.

## Electronic supplementary material

Supplementary Information

Temperature dependent deformation of 3D printed PNIPAAm structure

Gripping motion of 3D printed PNIPAAm structure fabricated with different levels of light intensity

Sequential deformation of 3D printed PNIPAAm structure by selective incorporation of ionic monomer
