# Supplementary material for: Micro 3D Printing of a Temperature-Responsive Hydrogel Using Projection Micro-Stereolithography
Source: Sci Rep. 2018 Jan 31;8:1963. doi: 10.1038/s41598-018-20385-2 (PMC5792483; doi:10.1038/s41598-018-20385-2)
Supplement: Supplementary file 1 — Supplementary Information [file 41598_2018_20385_MOESM1_ESM.doc]

**Supplementary Information**

**Micro 3D Printing of a Temperature-Responsive Hydrogel Using Projection Micro-Stereolithography**

**Daehoon Han^1^, Zhaocheng Lu^1^, Shawn A. Chester^2^, Howon Lee^1^***

^1^Department of Mechanical and Aerospace Engineering, Rutgers University, New Brunswick, NJ 08901, USA

^2^Department of Mechanical and Industrial Engineering, New Jersey Institute of Technology, Newark, NJ 07102, USA

*Corresponding author: howon.lee@rutgers.edu

**Simulation**

Thermally responsive gel behavior is analyzed in the context of the theoretical model in the recent study^1^, and for brevity, only the main points are repeated here. Since the experiment was performed over a very long time, equilibrium is assumed both mechanically and chemically, therefore inertia and diffusion are not considered. Further, since the body is not constrained, we consider stress free swelling, so that the deformation will be isotropic. Under these conditions, the model reduces to a nonlinear scalar equation for the swelling ratio


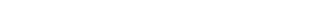
 (1)

Here, *ϕ* is the polymer volume fraction (a measure of swelling ratio), *R* is the gas constant, *θ* the absolute temperature, *Ω* the molar volume, *G* the shear modulus, and lastly the interaction parameter *χ* characterizes the interaction between the polymer and solvent. In the model, the key to the thermally responsive swelling is *χ*, which is based on the mixing contribution to the behavior of the material. The specific form chosen for this work comes from a recent study^1^, and has the form


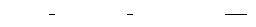
 (2)

Here, *χ_L_* is the value at a “low” temperature, *χ_H_* the value at a “high” temperature, *θ_T_* the transition temperature, and *Δ* the width of the transition. For calibration, we need to assume the shear modulus, and since we do not currently have reliable data, we have assumed the shear modulus is 0.5 MPa. Further, since the data shows a swelling ratio less than one at high temperatures, the structure was fabricated with some initial amount of fluid inside. We have estimated that initial swelling at *ϕ_0_*=0.3772 such that the polymer is 95% dry in the high temperature condition. Therefore, knowing *ϕ* from analysis of the experimental data, we are able to solve the nonlinear equation for *χ(θ)*, which is then calibrated as shown in the figure below, which fits very well.


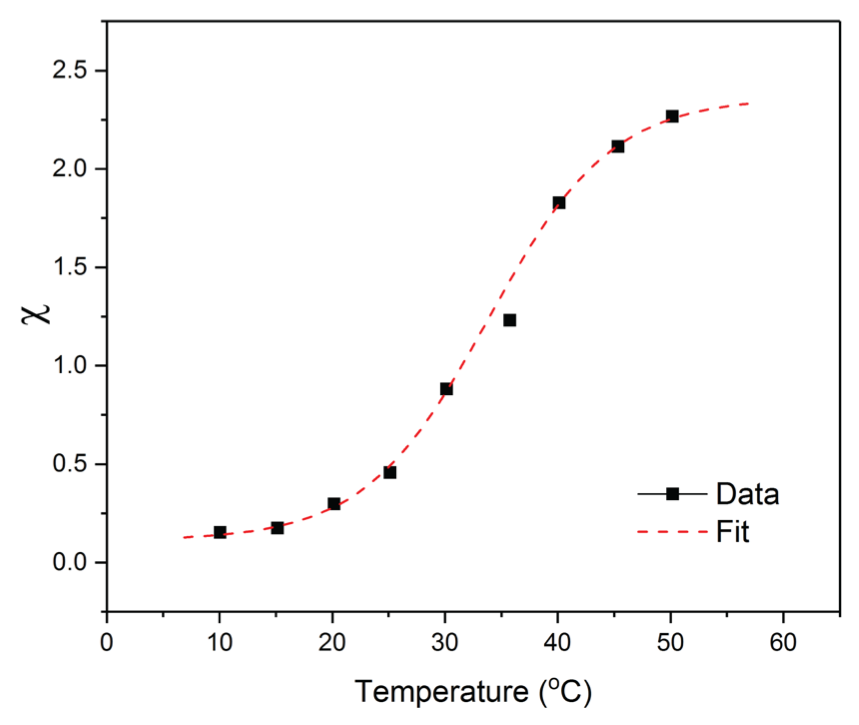


Next, with *χ(θ)* calibrated to the data for PNIPAAm, we are able to use a finite element procedure to simulate the behavior of various geometries and thermo-mechanical loading conditions. The full set of material parameters is given in below table.

| Parameter | Value |
| --- | --- |
| Gas constant | 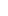 = 8.31457 J/(mol K) |
| Shear modulus | 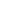 = 0.5MPa |
| Molar volume | 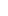 = 18.0695 cm^3^/mol |
| Interaction parameter at low temperature | 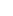 = 0.112 |
| Interaction parameter at high temperature | 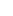 = 2.365 |
| Transition temperature | 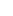 = 307K |
| Width of the transition | 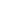 = 10.94K |

**Supplementary Table**

**Table S1**. Chemical composition of different polymer resins and the quantities of each component.

| Study | Chemical composition | | | | | | |
| --- | --- | --- | --- | --- | --- | --- | --- |
|  | Ethanol  (mL) | NIPAAm  (g) | Cross-linker  (g) | PI  (g) | PA  (g) | Rhodamine B  (g) | MAPTAC  (mL) |
| Curing depth  (Section 2.1) | 100 | 70 | 5 | 2 | 0.1 | 0.1 | - |
|  |  |  |  |  | 0.2 |  |  |
|  |  |  |  |  | 0.3 |  |  |
|  |  |  |  |  | 0.4 |  |  |
| Cross-linker  (Molar ratio)  (Section 2.2.1) | 100 | 70 | 1 | 2 | 0.3 | 0.1 | - |
|  |  |  | 2 |  |  |  |  |
|  |  |  | 3 |  |  |  |  |
|  |  |  | 4 |  |  |  |  |
|  |  |  | 5 |  |  |  |  |
| NIPAAm concentration  (Section 2.2.1) | 100 | 30 | 2.1  3.6  5.0  6.4  7.9 | 0.9 | 0.3 | 0.1 | - |
|  |  | 50 |  | 1.4 |  |  |  |
|  |  | 70 |  | 2.0 |  |  |  |
|  |  | 90 |  | 2.6 |  |  |  |
|  |  | 110 |  | 3.1 |  |  |  |
| Light intensity  (Section 2.2.2) | 100 | 70 | 5 | 2 | 0.3 | 0.1 | - |
| Layer thickness  (Section 2.2.3) | 100 | 70 | 5 | 2 | 0.3 | 0.1 | - |
| Ionic monomer, MAPTAC  (Section 2.2.4) | 100 | 70 | 5 | 2 | 0.3 | 0.1 | - |
|  |  |  |  |  |  |  | 4.2 |
|  |  |  |  |  |  |  | 8.4 |
| Gripper  (Section 2.3) | 100 | 70 | 5 | 2 | 0.3 | 0.1 | - |
| Dumbbell  (Section 2.3) | 100 | 70 | 4 | 2 | 0.3 | 0.1 | - |
|  |  |  | 5 |  |  |  | 8.4 |

**Table S2**. PμSL process parameters.

| Study | PμSL process parameters | | | |
| --- | --- | --- | --- | --- |
|  | Light intensity  (mW cm^-2^) | Curing time  (sec) | Layer thickness  (μm) | Number of layers |
| Molar ratio  (Section 2.2.1) | 30 | 10 | 30 | 10 |
| NIPAAm concentration  (Section 2.2.1) | 30 | 20 (for 2.6 M NIPAAm) | 30 | 10 |
|  |  | 15 (for 4.4 M NIPAAm) |  |  |
|  |  | 10 (for 6.2 M NIPAAm) |  |  |
|  |  | 8 (for 8.0 M NIPAAm) |  |  |
|  |  | 5.5 (for 9.7 M NIPAAm) |  |  |
| Light intensity  (Section 2.2.2) | 4.3  (Grayscale 100) | 4 | 30 | 10 |
|  | 7.3  (Grayscale 150) |  |  |  |
|  | 14.3  (Grayscale 200) |  |  |  |
|  | 30  (Grayscale 255) |  |  |  |
| Layer thickness  (Section 2.2.3) | 30 | 10 | 30 | 120 |
|  |  |  | 60 | 60 |
|  |  |  | 90 | 40 |
|  |  |  | 120 | 30 |
| Ionic monomer (MAPTAC)  (Section 2.2.4) | 30 | 10 | 30 | 10 |
| Gripper  (Section 2.3) | 4.3  (Grayscale 100) | 4 | 30 | 95 |
|  | 30  (Grayscale 255) |  |  |  |
| Dumbbell  (Section 2.3) | 30 | 10 | 30 | 154 |

**Supplementary Figures**


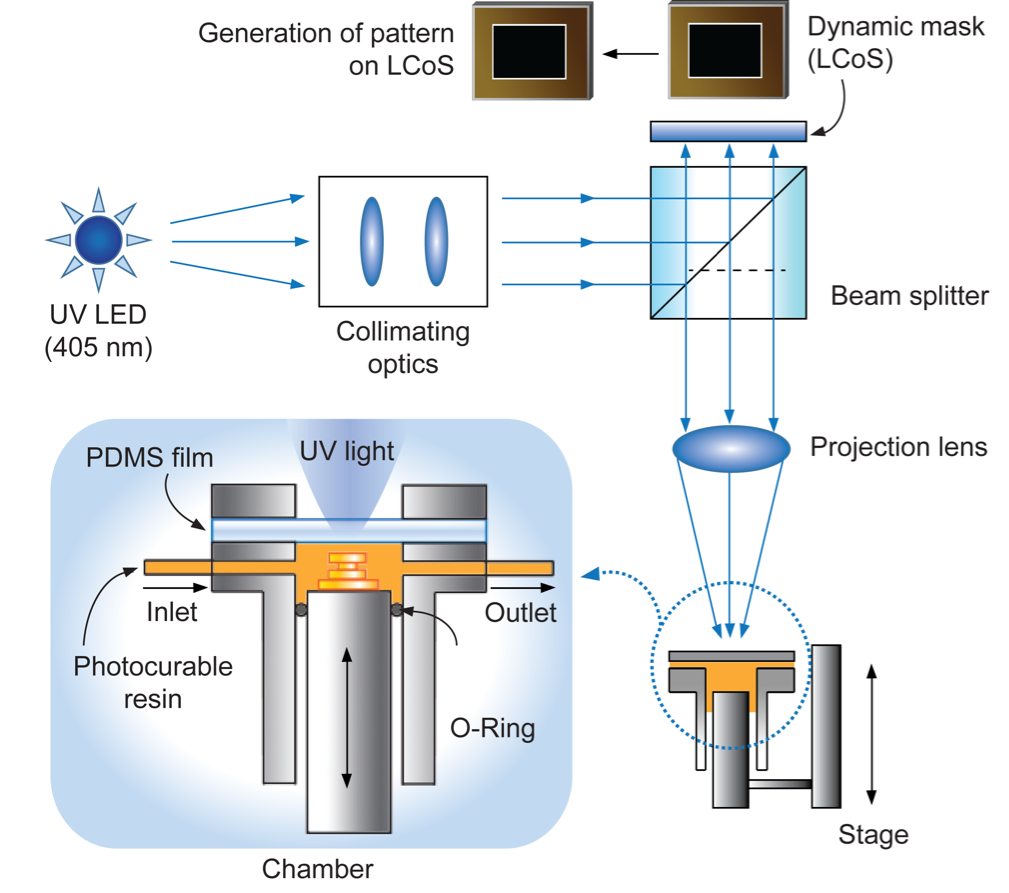


**Supplementary Figure S1.** Schematic drawing of the high resolution PμSL system.


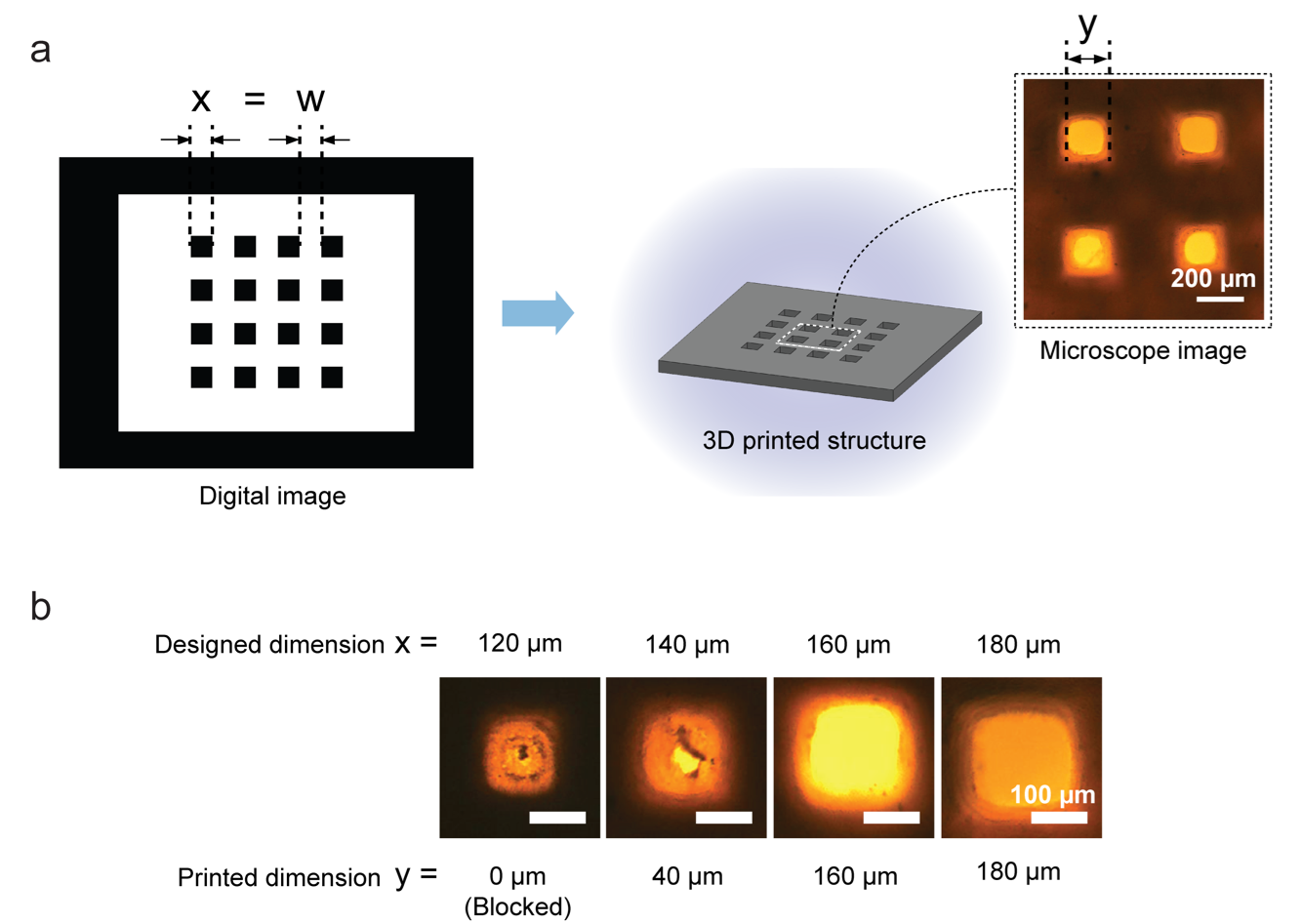


**Supplementary Figure S2.** Lateral resolution test for PNIPAAm 3D printing. (a) A digital image with multiple grid lines was projected to test the minimum possible feature size. The width of the grid lines (*w*) and that of holes (*x*) were identical in the digital image. The width of the holes in the printed structure (*y*) was measured using a microscope. (b) To determine the lateral resolution, four digital images with different width of holes and lines, 120, 140, 160, and 180 μm, were prepared and projected to create the structures. The printed hole sizes were 0, 40, 160, and 180 μm, respectively. The results show that the lateral resolution of PμSL with PNIPAAm hydrogel is around 160 μm.


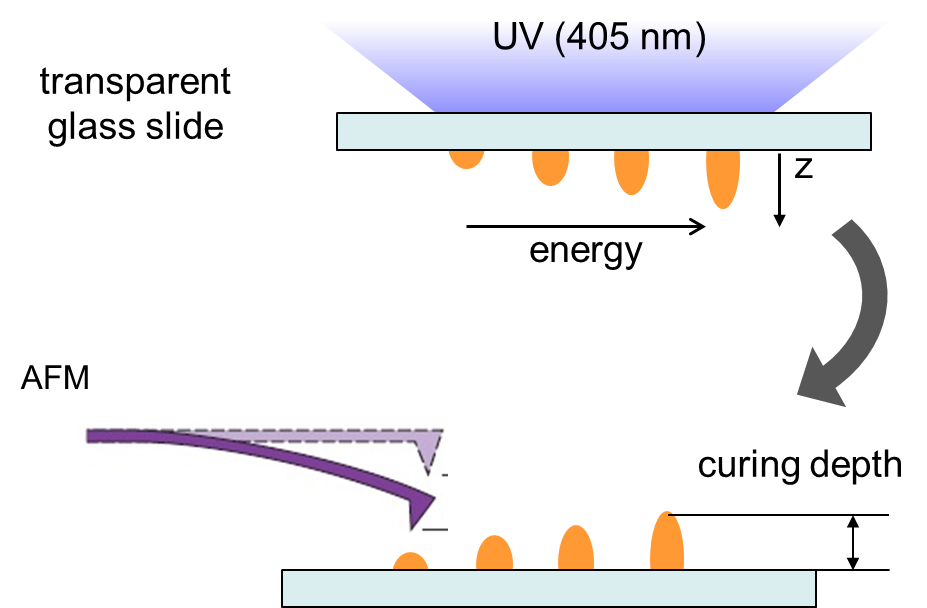


**Supplementary Figure S3.** Schematic drawing of the process used for the curing depth study using an AFM.


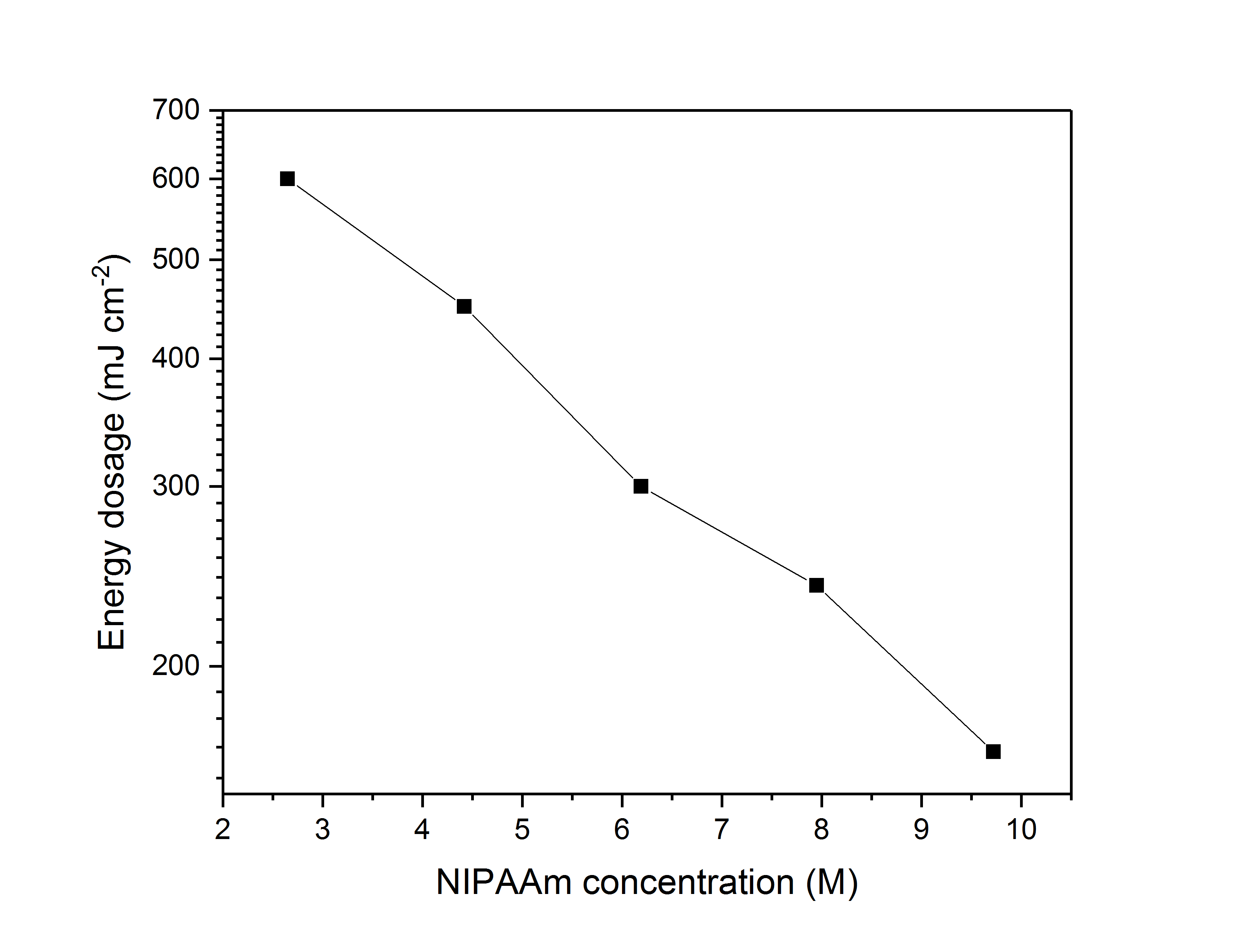


**Supplementary Figure S4.** Energy dose required to cure a 30 μm thick layer for different NIPAAm concentrations. An excessive energy dose results in unwanted polymerization.


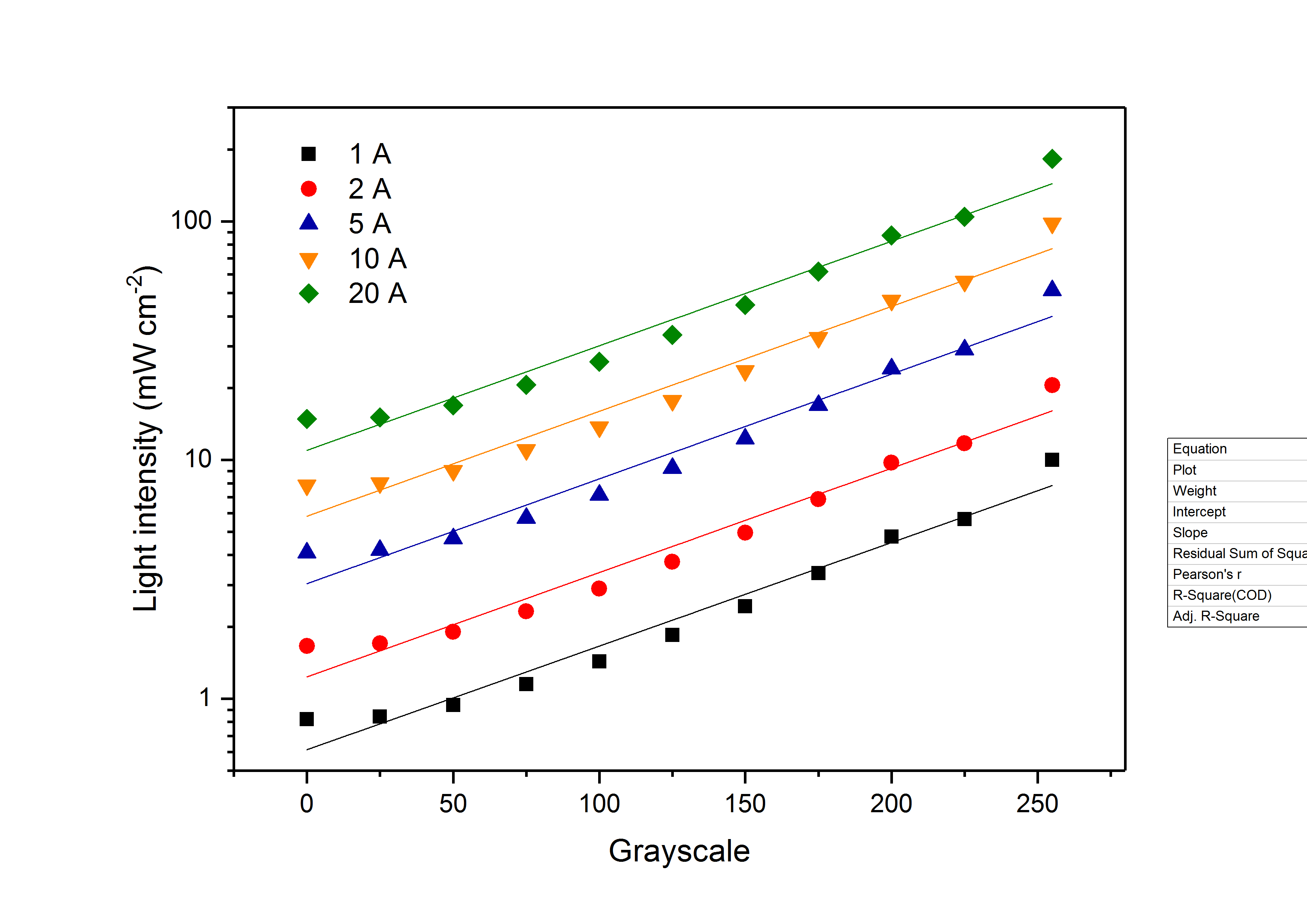


**Supplementary Figure S5.** The light intensity of the PμSL system for grayscales of a digital image and for different applied currents.


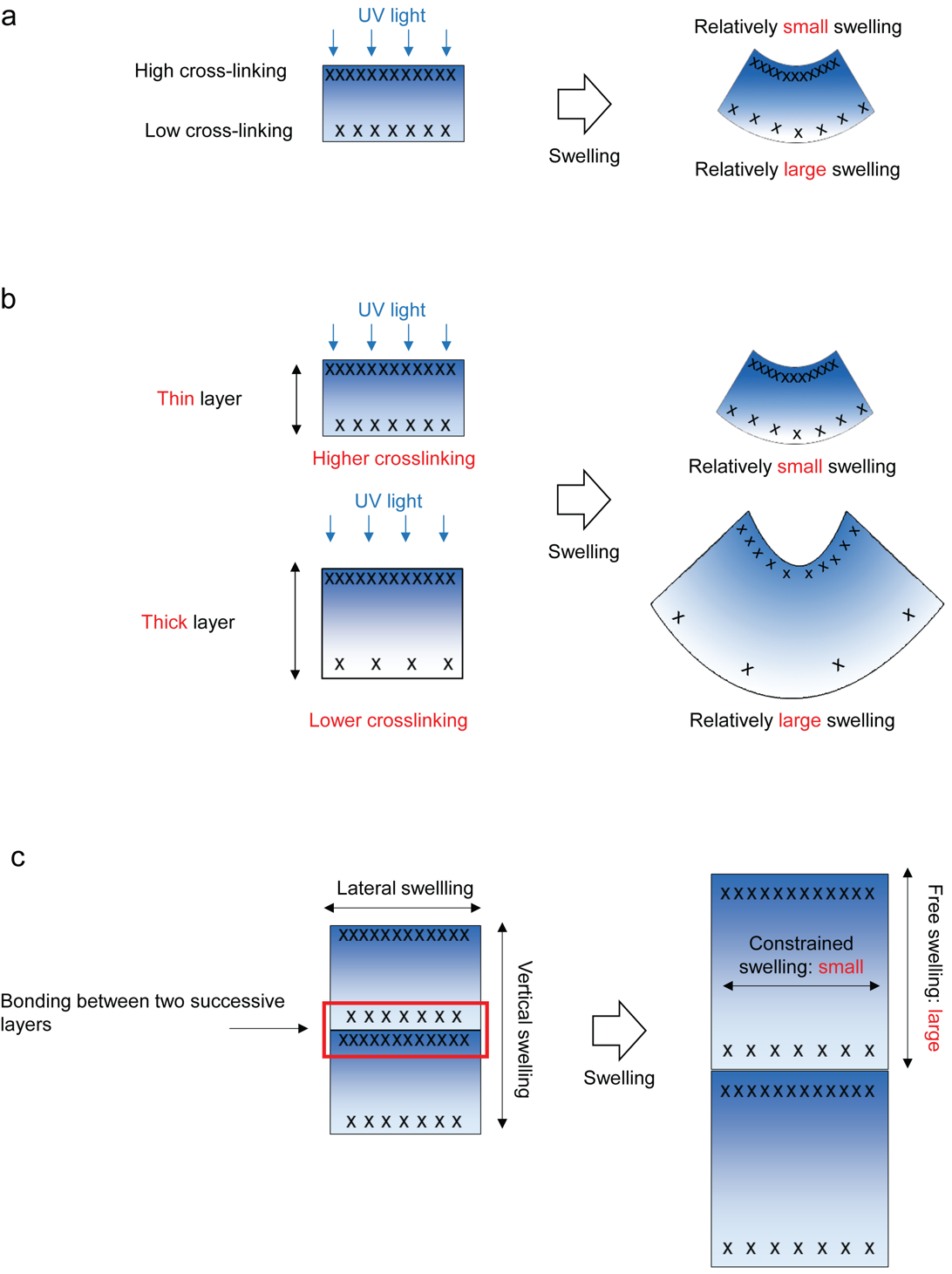


**Supplementary Figure S6.** The lateral and vertical swelling of 3D printed PNIPAAm layers. (a) A gradient of cross-linking density within a layer and corresponding swelling. (b) Since cross-linking density decreases with layer thickness, larger swelling is expected to occur at the bottom of the layer. (c) Bonding of successive layers constrains lateral swelling while swelling in a vertical direction occurs freely, resulting in overall swelling anisotropy.


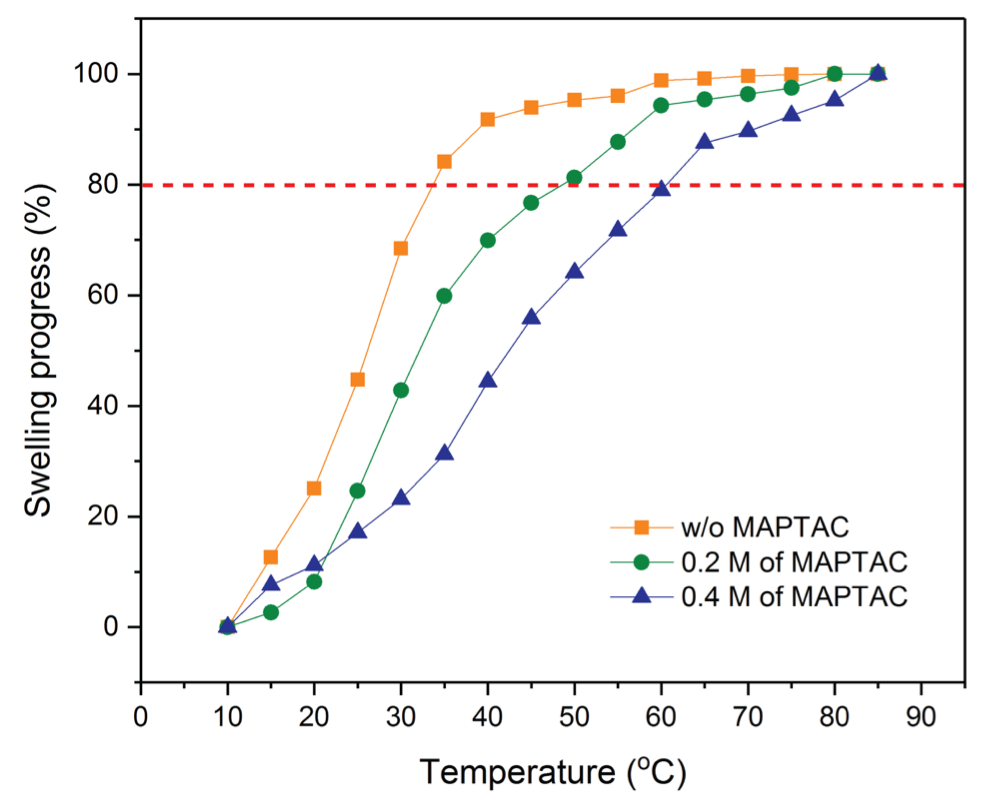


**Supplementary Figure S7.** Swelling progress as a function of temperature for various concentrations of MAPTAC. More than 80 % of the entire swelling ratio change was achieved at 35 °C without MAPTAC, at 50 °C with 0.2 M of MAPTAC, and at 65 °C with 0.4 M of MAPTAC. The progress was calculated with following equation:


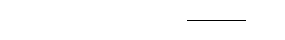


Here, *SR_t_* is the swelling ratio at specific temperature, and *SR_H_* and *SR_L_* are the swelling ratios at highest temperature (85 °C) and at lowest temperature (10 °C), respectively.

**Supplementary Video S1: Temperature dependent deformation of 3D printed PNIPAAm structure.** Video shows the swelling and shrinkage of 3D printed object in response to temperature.

**Supplementary Video S2: Gripping motion of 3D printed PNIPAAm structure fabricated with different levels of light intensity.** Video shows a gripping motion of 3D printed object when the temperature rises.

**Supplementary Video S3: Sequential deformation of 3D printed PNIPAAm structure by selective incorporation of ionic monomer.** Video shows a sequential shrinkage of 3D printed object that indicates the shifting of the transition temperature.

**Reference**

1 Chester, S. A. & Anand, L. A thermo-mechanically coupled theory for fluid permeation in elastomeric materials: application to thermally responsive gels. *Journal of the Mechanics and Physics of Solids* **59**, 1978-2006 (2011).
